# A Novel D-peptide modulates DCLK1 Gelsolin interactions, reducing PDAC tumor growth

**DOI:** 10.21203/rs.3.rs-6099914/v1

**Published:** 2025-03-11

**Authors:** Landon L. Moore, Dongfeng Qu, Parthasarathy Chandrekesan, Kamille Pitts, Randal May, Byron E. Anderson, Milton Brown, Courtney W. Houchen

**Affiliations:** University of Oklahoma Health Sciences Center; University of Oklahoma Health Sciences Center; University of Oklahoma Health Sciences Center; University of Oklahoma Health Sciences Center; University of Oklahoma Health Sciences Center; Independent Researcher; Macon & Joan Brock Virginia Health Sciences at Old Dominion University; University of Oklahoma Health Sciences Center

**Keywords:** DCLK1, pancreatic ductal adenocarcinoma (PDAC), Gelsolin, D-peptides

## Abstract

What drives inflammation-associated tumorigenesis and progression in pancreatic ductal adenocarcinoma (PDAC)? Doublecortin-like kinase 1 (DCLK1) is a central driver of inflammation-associated tumorigenesis, with elevated expression linked to worse clinical outcomes. Isoform 4, which lacks microtubule-binding domains but contains a unique extracellular domain (ECD), plays a pivotal role in tumor progression. We identified novel D-peptides that selectively target this ECD, significantly suppressing PDAC cell proliferation in vitro and tumor growth in xenograft models without inducing cell death. In silico modeling and binding assays revealed DCLK1 isoform 4 interacts with pro-tumorigenic proteins like plasma gelsolin (pGSN), with D-peptides modulating these interactions. These findings underscore DCLK1’s non-kinase functions as a therapeutic target and highlight novel avenues for developing precision treatments aimed at halting cancer progression and improving patient outcomes.

## INTRODUCTION

**Figure 1:**
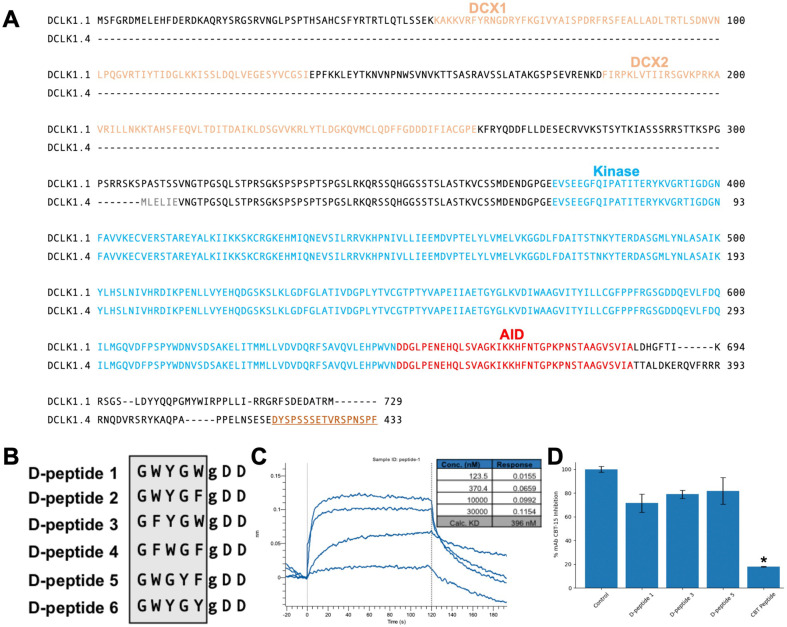
Isolation of D-peptides Targeting the Unique C-terminus of DCLK1 Isoform 4. **(A)** Alignment of human DCLK1 isoform 1 (α-long, NP_004725) and isoform 4 (β-short, NP_001182345) highlights structural differences. Isoform 1 contains the microtubule-binding domains DCX1 and DCX2 (orange), absent in isoform 4, along with the conserved kinase domain (red) and auto-inhibitory domain (blue). The peptide sequence used to develop CBT-15 is underlined. **(B)** Amino acid sequences of six D-peptides identified from the screen targeting the DCLK1 isoform 4 C-terminus. The gray box highlights the pentapeptide sequence forming the core of the D-peptide library. **(C)** Bio-layer interferometry (BLI) analysis shows binding of D-peptide 1 at four concentrations to DCLK1 isoform 4 immobilized on a biosensor to determine the K_D_ of 396 nM. **(D)** D-peptide 1 interacts at a site distinct from CBT-15’s binding site. A competition assay demonstrates CBT-15 binding to DCLK1. Data are presented as mean ± SEM; *p < 0.01.

Doublecortin-like kinase 1 (DCLK1) is a recognized marker of tumor stemness across many solid tumor cancers, including pancreatic ductal adenocarcinoma (PDAC).^1^ It plays a key role in epithelial-to-mesenchymal transition (EMT), facilitating metastasis and resistance to chemo-, radio-, and immunotherapies.^2–4^ Recent studies have identified crystal structures of the DCLK1 kinase domain, spurring interest in developing specific kinase inhibitors.^5^ However, DCLK1’s multi-functional nature, derived from its isoforms, DCLK1 isoform 1 (alpha-long) and DCLK1 isoform 4 (beta-short), adds complexity to understanding its precise roles in tumorigenesis.^6,7^ DCLK1 isoform 4 lacks the microtubule-binding domains but includes a unique extracellular domain (ECD) and is strongly associated with metastasis and poor survival (Fig. 1A).^8^

Crystal structure studies of the DCLK1 kinase domain that demonstrate binding to drug inhibitors show a complex regulatory relationship between the functional domains of DCLK1.^9,10^ Interestingly, the DCLK1 kinase domain is regulated by a C-terminal autoinhibitory domain (AID), the novel Hippocalcin like-1 protein (HPCAL1) binds to the AID and activates the DCLK1 kinase in a Ca^2+^-dependent manner.^11,12^ Thus, C-terminal protein-protein interactions (PPI) control DCLK1 kinase activity and may provide for more nuanced forms of regulation. We previously reported that a monoclonal antibody (CBT-15) targeting a unique ECD region, termed the non-kinase extracellular binding domain (NKEBD), reduces PDAC tumor growth and disrupts tumorigenic signaling.^7,13^ Here, we extend these findings by developing a novel D-peptide that binds the NKEBD of DCLK1 isoform 4, inhibiting tumor growth in vitro and in vivo. Additionally, we identify potential interacting proteins through in silico modeling, revealing mechanisms of DCLK1-mediated pro-tumorigenic activity.

## RESULTS

### Identification of D-peptides targeting the C-terminus of DCLK1 isoforms 2/4

Building on the success of CBT-15, we hypothesized that the unique C-terminus of DCLK1 isoform 4 has functional domains that can be targeted by smaller, more stable D-peptides. We screened a proprietary D-peptide library enriched in hydrophobic and aromatic residues for peptides that bound specifically to isoform 4, excluding those that bound isoform 1, and identified six candidates (Fig. 1B). Among these, D-peptide 1 exhibiting the highest binding affinity (Fig. 1C, K_D_ = 396 nM).

Given our previous findings that mAb CBT-15 inhibits PDAC tumor growth^7,13^, we evaluated whether these D-peptides binding interferes with CBT-15 binding to DCLK1 isoform 4, suggesting potential functional overlap. Binding assays focused on D-peptide 1, D-peptide 3 (which is sequence-similar), and D-peptide 5 (which is dissimilar yet functionally active). None of these D-peptides significantly inhibited CBT-15 binding, whereas the CBT-15 peptide effectively blocked it (Fig. 1D). These results indicate that the D-peptides interact with a distinct epitope on DCLK1 isoform 4 compared to mAb CBT-15.

### D-Peptide 1 Suppresses PDAC Cell Proliferation and Tumor Growth

**Figure 2:**
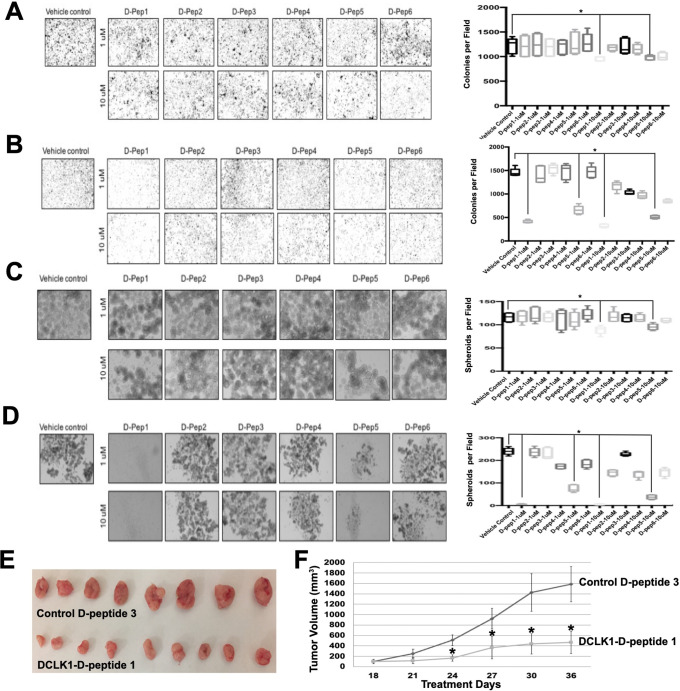
Anti-Tumor Effects of DCLK1 D-peptides on Pancreatic Cancer Cells **(A-B)** Colony Formation Assays: Boxplots show colony counts after treatment in **(A)** AsPC-1 and **(B)** Capan-1 pancreatic cancer cells. **(C-D)** PDAC Spheroid Self-Renewal: Boxplots display spheroid counts following treatment in **(C)** AsPC-1 and **(D)** Capan-1 cells. **(E-F)** In Vivo Efficacy of D-peptide 1: AsPC-1-derived xenografts were treated with D-peptide 1 or control peptide (D-peptide 3) every three days for six doses. **(E)** Representative tumor images after 36 days of treatment. **(F)** D-peptide 1 significantly reduced tumor size compared to control (Control n=8, D-peptide 1 n=9; *p<0.01).

Functional assays were conducted in PDAC cell lines AsPC-1 and Capan-1. Both D-peptides 1 and 5 significantly reduced colony formation at low micromolar concentrations (Fig. 2A–B). This blinded study revealed that D-peptides 2, 3, 4, and 6 were less active. Additionally, spheroid formation assays indicated that D-peptide 1 markedly impaired self-renewal capacity, suggesting a disruption of DCLK1-mediated stemness (Fig. 2C–D).

Given the inhibitory activity of D-peptide 1 in vitro, we investigated its effectiveness on tumor xenograft growth inhibition in vivo. Initial drug toxicity experiments in athymic mice found no apparent toxicity (Fig. S1). In athymic mice bearing AsPC-1 xenografts, D-peptide 1 treatment reduced tumor volume by 60% compared to controls (Fig. 2E–F). Importantly, tumor growth suppression occurred without inducing necrosis or apoptosis, indicating non-tumoricidal mechanisms.

### Identification of DCLK1 Binding Partners

**Figure 3:**
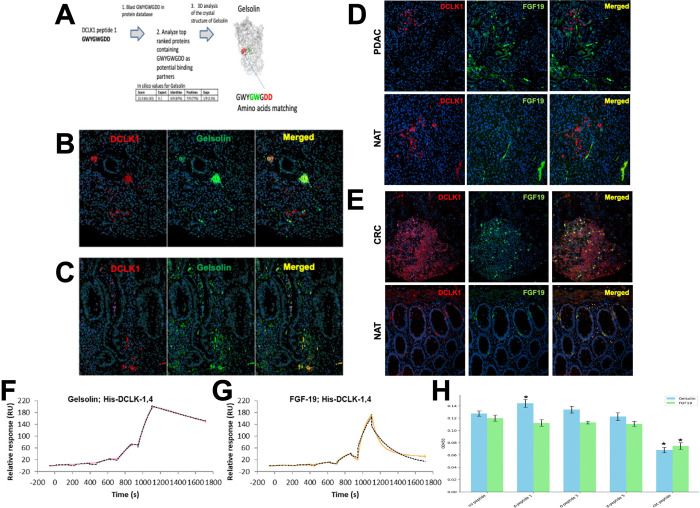
Gelsolin and FGF19 as Potential Extracellular DCLK1 Interactors **(A)** Identification Strategy for DCLK1 NKEBD Interactors: Step 1: BLAST peptide sequence against protein databases; Step 2: Analyze top-ranked proteins; Step 3: Upload and examine 3D structures; Step 4: Validate binding interactions experimentally. **(B-E)** Co-localization of DCLK1 with Gelsolin and FGF19: **(B)** DCLK1 and Gelsolin in PDAC; **(C)** DCLK1 and Gelsolin in CRC; **(D)** DCLK1 and FGF19 in PDAC; **(E)** DCLK1 and FGF19 in CRC. **(F-G)** SPR Binding Analyses: **(F)** DCLK1 isoform 4 binding to Gelsolin; **(G)** DCLK1 isoform 4 binding to FGF19. **(H)** Effects of Peptides on DCLK1-Gelsolin and DCLK1-FGF19 PPI.

Given the functional response of D-peptide 1 on tumor growth, we identified potential pro-tumorigenic binding partners that have the potential to bind specifically to the C-terminal extracellular domain of DCLK1. While the unique C-termini of DCLK1 isoform 4 and the related longer isoform DCLK1 isoform 2 are largely disordered and omitted from crystal structure-based analysis^9,14–16^, a high-quality candidate partial structure exists^17^ that can be modeled to obtain a representative structure for the DCLK1 NKEBD. Using this representative structure and a modeled structure for D-peptide 1 we looked for proteins with structures like D-peptide 1 that had known roles in cancer stemness, inflammation, immune modulation, or EMT. Based on the ECD amino acid sequence corresponding to the D-peptide 1 region we performed an in silico 3-D confirmational binding analysis with a stringency of greater than 80% binding affinity and identified five top potential binding partners (Table 1) Based on our 3-D modeling and in silico top hit strategy (Fig. 3A) we chose to evaluate the key innate immunity modulatory protein plasma gelsolin (pGSN) which is extracellularly localized and has also been implicated in the tumorigenic processes in several solid tumor cancers including PDAC.^18,19^ Immunohistochemistry revealed intermittent colocalization of DCLK1 and pGSN in PDAC tissues (Fig. 3B). However, in a cancer more affiliated with inflammation we found that the colocalization of DCLK1 and pGSN in colon cancer was more abundant. (Fig. 3C).

We also examined another candidate DCLK1 interacting protein known to be extracellularly localized, FGF19. In both PDAC and normal adjacent tissue (NAT) tissue, no colocalization was observed between DCLK1 and FGF19 (Fig. 3D). In contrast we observed that DCLK1 and FGF19 were colocalized in both colorectal cancer and NAT tissues (Fig. 3E) supporting a role for their interaction in colon cancers but not PDAC.

Given the colocalization results we examined whether DCLK1 isoform 4 physically interacts with pGSN and FGF19 using surface plasmon resonance (SPR). We found that PPI between DCLK1 isoform 4 and Gelsolin or FGF19 via SPR data indicated that DCLK1 isoform 4 is associated with FGF19 with a 1–1.8 mM K_D_, whereas Gelsolin was ~ 100 nM K_D_ (Fig. 3F, 3G, and S1).

### Biophysical Characterization of DCLK1-Gelsolin Protein-Protein Interactions

To assess the effects of DCLK1 NKEBD peptides on the binding of pGSN and FGF19 to DCLK1, we conducted ELISA-based binding assays. Initially, we observed consistent, linear binding between DCLK1 isoform 4 and pGSN across the gradient, with maximum binding at 2 μg/ml (Fig. S2). We used this concentration to test whether D-peptide 1, D-peptide 3, D-peptide 5, or the CBT-15 blocking peptide could disrupt pGSN or FGF19 binding to DCLK1. FGF19 bound to DCLK1 isoform 4 similarly to pGSN and the CBT-15 peptide effectively blocked their binding to DCLK1 isoform 4 (Fig. 3H, CBT peptide). Interestingly, the D-peptides, especially D-peptide 1, did not block the PPI interaction but seemed to improve pGSN binding to DCLK1 suggesting a potential synergistic effect (Fig. 3H, D-peptide 1). These results demonstrate that while pGSN and FGF19 interact with DCLK1 at the C-terminal ECD, the D-peptide 1 binding region is distinct from that of the CBT-15 epitope.

## DISCUSSION

This study identifies and characterizes a novel D-peptide, D-peptide 1, that targets the unique C-terminal region of DCLK1 isoforms 2 and 4 by focusing on the NKEBD rather than the kinase domain. This approach reveals an underappreciated role of the NKEBD in modulating PPIs involved in tumor progression. D-peptide 1 binds specifically to DCLK1 isoform 4 with minimal cross-reactivity to isoform 1. Competitive binding assays demonstrate that its binding site is distinct from that of mAb CBT-15, suggesting potential for combinatorial or sequential targeting strategies. In silico binding analyses, supported by SPR and ELISA assays, identified pGSN as a key binding partner of DCLK1 isoform 4, with a strong affinity (KD ~ 100 nM) indicative of a critical role in pro-tumorigenic signaling.^20,21^ Notably, D-peptide 1 enhances pGSN binding, implying allosteric modulation that may alter downstream signaling related to tumor growth and stemness, while its weak interaction with FGF19 underscores the selective nature of these PPIs.

Peptides have emerged as effective tools to disrupt PPI in cancer biology, thanks to their small size, conformational flexibility, and high specificity.^22,23^ Regulating PPI has been a longstanding goal in drug discovery^24^, and peptides’ small size and conformational flexibility enhance their attractiveness for this strategy. Therapeutic peptides disrupting PPIs face challenges like protease degradation,^25^ but advances in peptide chemistry have improved stability^26^. D-peptides, which resist degradation, offer promise for targeting PPIs.^27^ Compared to conventional L-peptides, D-peptides offer greater in vivo stability, specificity, and reduced immunogenicity, and can be readily modified for imaging, diagnostics, improved oral absorption, and tissue-specific targeting.^28–31^

Multiple binding partners interacting with distinct motifs in the C-terminal domain likely contribute to DCLK1’s roles in both inflammation and cancer. D-peptide 1 effectively suppressed tumor growth and stemness in vitro and in vivo potentially by modulating pGSN or other PPI binding without complete disruption, underscoring the therapeutic potential of targeting the DCLK1 NKEBD. Given the promising preclinical data, future studies should optimize D-peptide 1 for clinical application and further elucidate the impact of enhanced pGSN binding on tumor biology. Additionally, combinatorial regimens with agents like CBT-15 may refine therapeutic strategies targeting the multifaceted roles of DCLK1.

## METHODS

### Synthesis of D-peptide Library:

Solid-phase peptide synthesis was used to create the D-peptide probes using appropriate protection/deprotection steps.^32^ The D-peptide library was synthesized (Peptides International, Louisville, KY), using a TentaGel S resin, NH_2_ (TentaGel beads). Except for glycine, which is an achiral molecule, all the amino acid residues in the D-peptides are of the D-configuration. The TentaGel beads have a polystyrene core with polyoxyethylene arms attached to the core; each arm has a primary amine functional group at its terminus. The resin contains 8.8 7× 10^5^ beads/gram, an average bead diameter of 130 microns, 0.2–0.3 med/gram capacity, and 280–330 pmol of primary amine groups/bead capacity. The amino acids were conjugated to the resin and deprotected using standard D-peptide synthetic chemistries. Glycine was attached to the resin to achieve about a 30% substitution of the available primary amine groups at the ends of the polyoxyethylene chains of the TentaGel beads. The amine groups to which glycine was not added were blocked by acetylation using acetic anhydride. A 30% substitution yields an average spacing of about 100 to 200 angstroms between D-peptides on the bead surface. This was done to optimize the binding of a single protein to a single D-peptide sequence and to reduce the likelihood that steric hindrance will prevent a protein molecule from binding to a D-peptide or that a protein molecule will bind to more than one D-peptide.

Following the blocking of the unreacted primary amine groups, the D-peptide library was built by the split synthesis method.^33^ The resin mixture was divided equally into five portions and one of the amino acids, Glycine (G), D-Alanine (A), D-tryptophan (W), D-tyrosine (Y), or D-phenylalanine (F), was added by covalent coupling to each of the five portions of the G-substituted resin. The beads were then recombined, and again equally divided into five portions, and each used in reactions one of G, A, F, Y, or W. This procedure was repeated for five cycles to yield a library of pentapeptide sequences attached to the G residues of the resin. Each bead contained multiple copies of a single D-peptide sequence. Because five amino acids were used at each of the five amino acid-adding steps, the resulting bead library contained 3125 unique pentapeptide sequences. Following the final amino acid addition, the reaction mixtures were not recombined, which resulted in five sub-libraries of 625 different sequences, designated G, A, F, Y, or W, according to the last amino acid added. A sixth G amino acid residue was then added to all sub-libraries, before the addition of 2–3 amino acids with charged R groups, specifically aspartic acid (D) and lysine (K). This resulted in a total library of ~50,000 different D-peptide sequences.

### Screening the D-peptide library:

An aliquot from each sub-library, containing approximately 1000 beads, was added to a well of a 96-well polystyrene multi-well plate. From 1.5 to 2 ml of Superblock (Pierce Chemical Company, Rockford, IL.) reagent, 0.1% gelatin, or 1%(w/v) bovine serum albumin (BSA) in phosphate-buffered saline (PBS), pH 7.4, was added to each well, and the plates were incubated for one to two hours at room temperature (RT), with periodic or continuous mixing by gentle rocking. Purified DCLK1 isoform 4 protein was labeled with biotin using the biotinylating reagent NHS-LC-biotin (Pierce Chemical Company) according to the supplier’s instructions. Biotin-labeled protein was detected using AP-conjugated to neutravidin (Pierce Chemical Company) incubated with the beads for 30 minutes, after which the beads were washed three times with a Tris-buffered saline solution (pH 7.5), with the second wash being left in contact with the beads for 30 minutes. One-step NBT/BCIP (nitro-blue tetrazolium chloride/5-bromo-4-chloro-3’-indolyphosphate p-toluidine salt) (Pierce Chemical Co.) was then added and the beads observed under a low-power microscope until some of the beads had turned a dark purple color. The beads were washed with PBS twice, followed by 1% acetic acid and a final wash in water. Dark purple-black beads were removed using a small-bore pipette and subjected to amino acid sequence analysis.

### Synthesis and Purification of DCLK1 isoform 4:

Purification of human DCLK1 isoform 4 was performed at the University of Oklahoma Protein Production and Characterization Core Facility (Norman, OK) using an *E. coli* strain BL21(DE3) pLPP containing the pET19-DCLK1 isoform 4 plasmid. For Bio-layer Interferometry (BLI) experiments, a C-terminal DCLK1 isoform 2/4 His-tagged protein was used (NovoPro, Shanghai, China). All His-tagged proteins were dialyzed using PBS/glycerol to remove imidazole.

### BLI evaluation of D-peptide binding to DCLK1 NKEBD:

Human His-tagged DCLK1 C-terminal recombinant protein from NovoPro was used. Binding to the sensor was carried out in PBS/0.05%Tween20/1%DMSO. Response is measured as a nm shift in the interference pattern and is proportional to the number of molecules bound to the biosensor’s surface. Initially, all peptides were tested at 5 and 30 μM. The top binders were tested again with eight different concentrations, starting at 30 μM and diluted in a 1:3 series to determine the dissociation constant (KD).

### Proliferation Assay:

AsPC-1 and CAPAN-1 cells were grown as previously described with varying concentrations of candidate D-peptides.^34^ Afterward, we treated cells with TACS MTT Reagent (RND Systems) at 37C until a dark crystalline precipitate within the cells was observed. NH_4_OH in DMSO was added and incubated for 10 minutes while protected from light. We determined the OD_550_ and averaged it as a percentage of cell proliferation.

### Self-renewal Assay:

AsPC-1 and CAPAN-1 cancer cells were treated with candidate D-peptides or vehicle control and suspended in growth factor-reduced Matrigel^™^ for 7 days or 0.3% agar for 3 weeks and monitored for spheroid growth. The size and number of spheroids were analyzed, counted, and then compared between the treatment groups.^35,36^

### Ethical Statement:

The animal studies were approved by the University of Oklahoma Health Sciences Institutional Animal Care and Use Committee (IACUC) and were performed in accordance with all relevant guidelines and regulations. In addition, this study adheres to the ARRIVE guidelines for reporting research involving live animals.^37^ All anesthesia and euthanasia methods employed are consistent with current veterinary best practices.

### Mice Xenografts:

Athymic nude (*Foxn1*^*nu*^*/Foxn1*^*nu*^) mice were purchased from the Jackson Laboratory (Bar Harbor, Maine) and housed in pathogen-free conditions. AsPC-1 cells (1 × 10^7^) were injected subcutaneously into the flanks of 4- to 6-week-old mice (n=3/group). Tumors were measured using a caliper and the volume was calculated as (length × width^2^) × 0.5. Once tumors achieved an average size of ~100 mm^3^, mice were injected subcutaneously with selected D-peptides at a dose of 10 mg/kg. Treatments were continued every three days until day 36.

### ELISA assay:

Polystyrene microtitre Immulon 2HB plate was coated with freshly prepared (400 ng/50 ml) DCLK1 isoform 4 (50 ml/well) in 0.1 M NaHCO_3_, pH 8.6, overnight at 4C. After blocking with 1% gelatin in TTBS at RT, different concentrations of gelsolin or FGF19 were added (2 mg/50ul/well, 1 mg/well, 0.5 mg/well, and 0.25 mg/well at 50 ml/well, proteins diluted in TTBS), and incubated at RT. To study the competitive ability of peptides, we used 20 mg/well of either D-peptide 1, D-peptide 3, D-peptide 5, or the C-terminal peptide antigen for the mAb CBT-15. After incubation at RT, plates were washed with TTBS, and bound gelsolin or FGF19 was detected. Bound plasma gelsolin (ab233671; Abcam, Waltham, MA) was immunodetected with anti-gelsolin Ab (ab11081; Abcam, Waltham, MA) or FGF19 (969-FG-025; Bio-Techne, Minneapolis, MN) immunodetected by anti-FGF19 (sc-390621; Santa Cruz Biotechnology, Dallas, TX). Detection was by alkaline phosphatase-conjugated goat anti-mouse IgG (ab97020; Abcam, Waltham, MA) followed by 1-STEP NBT/BCIP substrate (#34042; ThermoFisher Scientific, Waltham, MA) and quantified at 450 nm on a microplate reader.

### Modeling of the peptide binding interaction with DCLK1 NKEBD:

We modeled peptide binding interactions with DCLK1 NKEBD using the high-quality partial structure 3VSM_A (100% identity, 0% gaps).^17^ While this structure provides a representative model for DCLK1 NKEBD, peptide configurations and binding conformations remain undefined and require computational modeling. The D-peptide 1 sequence is present in other proteins with resolved tertiary structures. A PDB search identified several relevant structures, including the X-ray structure of calcium-free human gelsolin (PDB: 3FFN_A).^38^ We used Autodock^39^ and ICM-Dock (Molsoft LLC, San Diego, CA) to predict ligand binding affinities, allowing ligand flexibility but assuming a rigid receptor. To address receptor flexibility, we employed docking platforms such as GOLD (The Cambridge Crystallographic Data Centre, Cambridge, UK), CABS-Dock^40^, and FlexAID^41^. Candidate conformations were identified through clustering based on pose, configuration, and predicted binding energy, enabling molecular dynamics (MD) simulations. We further applied free energy perturbation (FEP) methods based on MD simulations to estimate ligand binding affinities.^42^

### Statistical analysis:

For statistical analyses, we used either a student t-test or analysis of variance (ANOVA) for continuous outcomes or the Kruskal-Wallis test for the number of spheroids using GraphPad Prism, with P values <0.05 (two-sided) considered statistically significant. Data were transformed as necessary. All experiments were performed independently a minimum of three times. If the overall statistical significance was reached, pairwise comparisons (e.g., DCLK1 D-peptide vs. control D-peptide) were conducted with multiple tests adjusted by Bonferroni’s method.

## Figures and Tables

**Table 1:** Top Candidate DCLK1 NKEBD Interactors

Protein	Function	% Identity	Reference
SetD2	Methyltransferase for histones and microtubules, transcriptional regulation, genomic stability, and cytoskeletal functions	100	Park et al., (2016)^43^
FGF19	Proliferation, apoptosis resistance, and metastasis	100	Lang et al. (2019)^44^
Dvl2	Metastasis and chemoresistance	100	Yang et al. (2020)^45^
Gelsolin	Actin capping. Cytoskeleton regulation, and EMT	67	Zhang et al., (2020)^46^
Perforin	Pore formation, apoptosis, and immune suppression	60	Dufait et al., (2019)^47^

## Data Availability

All data are available in the main text or the supplementary materials.
